# Hippocampal tau‐induced *GRIN3A* deficiency in Alzheimer's disease

**DOI:** 10.1002/2211-5463.13904

**Published:** 2024-10-13

**Authors:** Sang‐Eun Lee, Soomin Park, Rian Kang, Taehoon Lee, Won Jong Yu, Sunghoe Chang, Jong‐Chan Park

**Affiliations:** ^1^ Department of Physiology and Biomedical Sciences, College of Medicine Seoul National University Korea; ^2^ Neuroscience Research Institute Seoul National University Medical Research Center Korea; ^3^ Department of Biophysics Sungkyunkwan University Suwon Korea; ^4^ Institute of Quantum Biophysics Sungkyunkwan University Suwon Korea; ^5^ Department of Metabiohealth Sungkyunkwan University Suwon Korea

**Keywords:** Alzheimer's disease, *GRIN3A*, hippocampus, NR3A, tau, transcriptome

## Abstract

Alzheimer's disease (AD) is characterized by significant alterations in hippocampal function and structure, but the molecular mechanisms underlying the hippocampal region remain elusive. We integrated multiple transcriptome datasets including human or rat hippocampus (GSE173955, GSE129051, GSE84422) to identify candidate genes. Subsequent analyses including gene ontology analysis and protein–protein interaction mapping were performed to identify key genes and pathways. We found that glutamate ionotropic receptor NMDA‐type subunit 3A (*GRIN3A*) and glutamate metabotropic receptor 8 (*GRM8*), which are related to the glutamatergic system, were the top two annotated genes and directly related to *MAPT*, which encodes a tau protein. Since there is no direct evidence of interaction between tauopathy and these genes in AD, further transcriptomic data (GSE125957, GSE56772) from tau transgenic mice and experimental validations through primary rat hippocampal neurons and induced pluripotent stem cell (iPSC)‐derived brain organoids were performed. Interestingly, we identified that decreased NR3A (encoded by *GRIN3A*) and mGluR8 (encoded by *GRM8*) are correlated with tauopathy and loss of postsynaptic function in AD. Taken together, our results identified a novel tauopathy biomarker *GRIN3A* in AD. Furthermore, our findings suggest that an integrated approach combining public databases and diverse experimental validations can contribute to the advancement of precision medicine therapies.

## Graphical abstract

Alzheimer's disease is characterized by alterations in hippocampal function, but the molecular mechanisms underlying these changes remain elusive. Through comparison of multiple transcriptome datasets, followed by gene ontology analysis and protein–protein interaction mapping, we identified *GRIN3A* and *GRM8* as the top two genes related to loss of postsynaptic function and tauopathy. Further experimental validation was also performed.

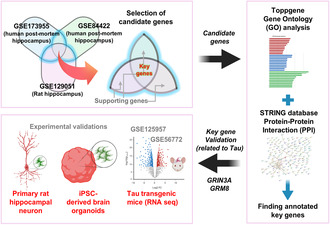

**Abbreviations**
AD: Alzheimer's disease
BP: biological process
CC: cellular component
CN: cognitively normal
CNS: central nerve system
FDR: false discovery rate
GEO: Gene Expression Omnibus
GO: Gene ontology
GPCR: G protein‐coupled receptor
GRIN3A: glutamate ionotropic receptor NMDA‐type subunit 3A
GRM8: glutamate metabotropic receptor 8
MF: molecular function
NMDA: *N*‐methyl‐d‐aspartate
PPI: protein–protein interaction
UMAP: uniform manifold approximation and projection

Alzheimer's disease (AD) is a progressive neurodegenerative disorder characterized by cognitive decline, memory loss, and functional impairment. It represents the most common cause of dementia in the elderly, posing significant challenges for individuals, families, and healthcare systems globally [1]. The pathological hallmarks of AD include amyloid beta plaques, neurofibrillary tangles, and widespread neuronal loss, particularly in regions critical for memory and cognition. As the global population ages, the prevalence of AD is expected to rise dramatically, making it imperative to develop effective strategies for diagnosis, treatment, and prevention [2].

The hippocampus, a key brain structure involved in learning and memory, is particularly vulnerable to the pathological processes of AD [3]. This region undergoes significant atrophy and functional impairment early in the disease course, making it a focal point for understanding AD pathology and progression [4]. Investigating the hippocampus provides critical insights into the mechanisms driving cognitive deficits and offers potential targets for therapeutic intervention. Understanding the specific molecular changes in the hippocampus is essential for developing targeted therapies to mitigate or halt AD progression [5].

Recent studies have started to shed light on the role of NR3A (encoded by *GRIN3A*), a subunit of the *N*‐methyl‐d‐aspartate (NMDA) receptor, in the context of AD [6, 7]. NMDA receptors are crucial for synaptic plasticity and memory function, and dysregulation of these receptors has been implicated in various neurodegenerative disorders, including AD. Specifically, NR3A has been shown to play a role in synaptic signaling and neuroprotection. Research indicates that alterations in NMDA receptor subunits, including NR3A, may contribute to the synaptic dysfunction observed in AD [6, 7]. NR3A is known to influence synaptic plasticity by modulating NMDA receptor activity, and its dysregulation could lead to impaired synaptic function, a hallmark of AD [6]. However, the exact mechanism by which NR3A contributes to the pathophysiology of AD remains largely unexplored. Moreover, although they induced GRIN3A knockout to modulate pathogenesis of AD, they did not confirm the direct evidence that amyloid beta plaques or tau, major pathological hallmarks of AD, can affect NR3A levels and its postsynaptic activity. Additionally, it is not well understood how NR3A interacts with other molecular pathways implicated in AD. This gap in knowledge underscores the necessity for further investigation to elucidate the specific role of GRIN3A in AD. Understating whether and how NR3A dysregulation contributes to the progression of AD could provide new therapeutic targets, potentially leading to the development of treatments that can restore normal NMDA receptor function and synaptic plasticity. In terms of synaptic transmission and plasticity in the brain, metabotropic glutamate receptor 8 (mGluR8), encoded by the *GRM8* gene, also can modulate neurotransmission indirectly through G protein‐coupled receptor pathways [8]. Although they are components of different receptor systems, they can intersect each other in the broader context of glutamatergic synaptic signaling and maintain the balance of excitatory neuronal functions [9]. Recent studies suggest that mGluR8 has a neuroprotective effect by modulating synaptic plasticity and reducing excitotoxicity, which are critical risk factors in AD [10, 11]. However, since there is limited evidence that the dysfunction of mGluR8 could contribute to the accumulation of amyloid plaques, more studies are needed to fully understand the precise mechanisms of mGluR8 and its relationship with NR3A in the pathogenesis of AD.

Given the complexity of AD, it is crucial to employ comprehensive and high‐throughput approaches to identify key molecular players and pathways involved in its pathogenesis [12]. Transcriptome analysis, which examines the complete set of RNA transcripts produced by the genome, offers a powerful tool for uncovering gene expression changes associated with AD [13]. Integrating data from multiple transcriptome studies is particularly important as it allows for identifying candidate genes and pathways that are consistently altered in AD across different populations and conditions [14]. This approach enhances the robustness of the findings and increases the likelihood of identifying true molecular targets that play a pivotal role in the disease process.

In this study, we employ an integrative approach to analyze transcriptome data from multiple datasets (GSE173955, GSE129051, GSE84422) to identify key genes involved in AD (https://www.ncbi.nlm.nih.gov/geo/). Our workflow includes the intersection of these datasets to pinpoint candidate genes, followed by gene ontology (GO) analysis using ToppGene (https://toppgene.cchmc.org/) to discover key pathways. We further mapped protein–protein interactions (PPI) using the STRING database to understand the functional interactions among the identified proteins. Validation of key genes (*GRIN3A*, *GRM8*) was performed using transcription data (GSE125957, GSE56772) from Tau transgenic mice, widely used models for studying AD, since we found significant interactions between tau and our key genes. Additionally, we performed imaging analysis of primary rat hippocampal neurons and patient‐derived induced pluripotent stem cell (iPSC)‐derived human brain organoids (postsynaptic structures and gene expression) to validate our findings. In summary, by leveraging diverse transcriptome datasets and experimental validations through diverse biological samples (human, rat, mouse samples, and iPSC‐derived brain organoids), our approach will ensure a more comprehensive and robust identification of critical hippocampal genes and pathways implicated in AD.

## Materials and methods

### GEO database and RNA sequencing analysis

To analyze transcriptome data from publicly available databases, we accessed NCBI Gene Expression Omnibus (GEO) (https://www.ncbi.nlm.nih.gov/geo/), a public functional genomics data repository. We performed GEO2R, an interactive web tool, to compare two groups and get differentially expressed genes (DEGs) from multiple datasets (GSE173955, GSE129051, GSE84422). We integrated transcriptome data to identify candidate genes by finding the intersection of these datasets. Visualizing volcano plots, uniform manifold approximation and projection (UMAP) graphs, and boxplots were done by GEO2R. We also used other datasets (GSE125957, GSE56772) for validation of key genes and supporting genes.

### GO analysis

We employed the ToppGene platform‐based gene ontology (GO) analysis (https://toppgene.cchmc.org/) to find relevant pathways to key and supporting genes. To obtain relevant pathways and targets from the candidate key and supporting genes (154 genes) which were overlapped in Venn diagram between multiple datasets (GSE173955, GSE129051, GSE84422), ToppFun function was performed with false discovery rate (FDR)‐corrected *P*‐values (*P* < 0.1). Molecular function (MF), biological process (BP), cellular composition (CC), and computational network terminologies were extracted from the ToppFun function. For the selected key gene (GRIN3A) and supporting genes (*ENO2*, *NPTXR*, *GUCY1A2*, *NME1*, *MAP2K4*, *GRM8*, *HTR5A*, *KCNJ8*) with *MAPT* gene (total 10 genes), ToppFun function was performed with FDR‐corrected *P*‐values (*P* < 0.05). MF, BP, CC, and disease terminologies were extracted from the ToppFun function.

### STRING database for PPI

We employed the STRING database for PPI analysis, an analytic tool for functional protein association networks (https://string‐db.org/). The candidate key and supporting genes (153 genes) with 7 AD signature genes (*APOE*, *MAPT*, *APP*, *PSEN1*, *PSEN2*, *TREM2*, *NEFL*) were input for the STRIND PPI analysis. We prioritized specific PPI which is linked with *GRIN3A* (the only overlapped gene between multiple datasets from GEO) and the relevant genes were sorted as the selected key and supporting genes (9 genes including *GRIN3A*).

### Animals

Animal experiments were approved by the Institute of Animal Care and Use Committee (IACUC, Approval ID number: SNU‐100930‐5) of Seoul National University, Korea. All experiments were carried out following approved guidelines and regulations.

### DNA constructs and antibodies

Plasmids encoding pRK‐EGFP‐Tau and pRK‐EGFP‐Tau P301L were kind gifts from Prof Inhee Mook‐Jung (Seoul National University, Seoul, South Korea). Primary antibodies used in this study are anti‐NMDAR3A (rabbit polyclonal, Alomone, AGC‐030, Jerusalem, Israel), anti‐mGluR8 (rabbit polyclonal, Alomone, AGC‐028), anti‐PSD95 (mouse monoclonal, Invitrogen, Carlsbad, CA, USA, MA1‐045), anti‐GAPDH (rabbit polyclonal, Abcam, ab9485, Cambridge, UK), anti‐MAP2 (goat polyclonal, Phosphosolutions, 1099‐MAP2t, Aurora, CO, USA), anti‐Phospho‐Tau (Thr181) (mouse monoclonal, ThermoFisher, MN1050, Waltham, MA, USA) and DAPI (Sigma‐Aldrich, D9542, Saint Louis, MO, USA), Alexa Fluor™‐647/568/405 labeled secondary antibodies were purchased from Thermo Fisher Scientific (Waltham, MA, USA).

### Primary neuron culture and transfection

Primary rat hippocampal neurons derived from embryonic day 18 Sprague Dawley fetal rats of either sex were prepared as described previously [15]. Briefly, hippocampi were dissected, dissociated with papain (Worthington Biochemical Corporation, Lakewood, NJ, USA), and resuspended in minimal Eagle's medium (MEM, Invitrogen, Waltham, MA, USA) supplemented with 0.6% glucose, 1 mm pyruvate, 2 mm l‐glutamine, and 10% fetal bovine serum (Hyclone, South Logan, UT, USA), and plated on poly‐d‐lysine‐coated glass coverslips in 60 mm Petri dishes. Four hours after plating, the medium was replaced with a neurobasal medium (Invitrogen) supplemented with 2% B‐27 (Invitrogen), 0.5 mm l‐glutamine. Neurons were transfected by a modified calcium phosphate method as previously described [15]. Briefly, 6 μg of DNA and 9.3 μL of 2 m CaCl_2_ were mixed in distilled water to a total volume of 75 μL and the same volume of 2× BBS [50 mm BES, 280 mm NaCl, and 1.5 mm Na_2_HPO_4_ (pH 7.1)] was added. The cell culture medium was completely replaced by transfection medium (MEM; 1 mm sodium pyruvate, 0.6% glucose, 10 mm HEPES, 1 mm Kynurenic acid, and 10 mm MgCl_2_, pH 7.71), and the DNA mixture was added to the cells and incubated in a 5% CO_2_ incubator for 60 min. Cells were washed with a washing medium (pH 7.30) and then returned to the original culture medium. Neurons were transfected at days *in vitro* (DIV) 8–9 and analyzed at DIV 19–21.

### Immunocytochemistry

Primary neurons were fixed for 15 min at room temperature (RT) in 4% (w/v) PFA, 4% (w/v) sucrose in PBS, pH 7.4 and subsequently permeabilized with 0.25% Triton X‐100 in PBS for 3 min at RT. The cells were then blocked for 1 h at RT in 10% (w/v) bovine serum albumin (BSA). Cells were incubated at 4 °C overnight in primary antibodies after which the cells were washed in PBS and incubated with secondary antibodies for 1 h at RT.

### High‐resolution imaging and analysis

Super‐resolution synapse images were acquired on a Zeiss LSM 980 microscope with Airyscan detector using a 63×, 1.4 NA oil immersion Plan‐Apochromat objective (Carl Zeiss, Oberkochen, Germany). A zoom factor of 3× and frame size of 1272 × 1272 was used for all images, resulting in an XY pixel size of 35.2 nm. The z‐step size was 130 nm, with 15 steps per Z‐stack. Scan speed was 5, gain 800, and digital gain 1. A 5%, 3%, 2%, and 2% laser power was used for the 405, 488, 561 nm, and 647 lasers, respectively. The Airyscan detector was aligned before imaging. Imaris software (Bitplane AG, Zurich, Switzerland) was used for NR3A and mGluR8 puncta detection. Channel brightness was adjusted to maximize the visualization of the immunoreactive region, and the surface function was then used to generate volumes representing NR3A and mGluR8. The colocalization of NR3A and mGluR8 with PSD95 were calculated for every z‐step using JACoP plug‐in in fiji (NIH software, Bethesda, MD, USA).

### Generation of induced pluripotent stem cell (iPSC)‐derived brain organoids

To examine the effect of paired helical filament (PHF)‐tau on human brain samples, human induced pluripotent stem cell (iPSC)‐derived cortical organoids were generated according to our previous reports [16, 17]. A commercialized iPSC (BIONi010‐C) from the European Bank for induced pluripotent Stem Cells (EBiSC) was used for this study. Briefly, embryoid bodies (EBs) were generated in AggreWell EB Formation Medium (ST05893, Stemcell Technologies, Vancouver, Canada) on AggreWell plate 800 (34 811, Stemcell Technologies). From day 2 to day 5, EBs maintained with DMEM/F‐12 containing GlutaMAX (10565‐018, Gibco), 20% KnockOut Serum Replacement (A3181501, Gibco, Waltham, MA, USA), 1% MEM Non‐Essential Amino Acids Solution (11140050, Gibco), 0.1 mm 2‐mercaptoethanol (21985023, Gibco), 100 U·mL^−1^ penicillin and 100 μg·mL^−1^ streptomycin (P4333, Merck, Darmstadt, Germany), dorsomorphin (10 μm; P5499, Merck), and SB‐431542 (10 μm; 1614, TOCRIS, Bristol, UK). From day 6 to day 24, EBs were transferred to ultra‐low‐attachment plates (7007, Corning, Corning, NY, USA) and cultured with Neurobasal‐A Medium (10888‐022, Gibco), B‐27 Supplement minus vitamin A (12587010, Gibco), 100 U·mL^−1^ penicillin and 100 μg·mL^−1^ streptomycin, GlutaMAX (35050‐061, Gibco), 0.5% (v/v) Matrigel basement membrane matrix (354234, Corning), 20 ng·mL^−1^ epidermal growth factor (01‐107, Merck), and 20 ng·mL^−1^ fibroblast growth factor basic (233‐FB, R&D Systems, Minneapolis, MN, USA). From day 25 to day 42, organoids were cultured with Neurobasal‐A Medium (10888‐022, Gibco), B‐27 Supplement minus vitamin A (12587010, Gibco), 100 U·mL^−1^ penicillin and 100 μg·mL^−1^ streptomycin, GlutaMAX (35050‐061, Gibco), 0.5% (v/v) Matrigel basement membrane matrix (354234, Corning), brain‐derived neurotrophic factor (450‐02, Peprotech, Cranbury, NJ, USA), and 20 ng·mL^−1^ neurotrophin‐3 (450‐03, Peprotech). From day 43, the matured organoids were maintained without any growth factors. On day 140, the organoids were used for performing immunohistochemistry.

### Western blot

The western blot (WB) experiment was performed to validate the NR3A levels in the brain organoids, according to our previous study [17, 18]. Briefly, the organoids were lysed with RIPA lysis buffer containing a protease/phosphatase inhibitor cocktail. Total proteins were quantified by BCA assay and equal quantities of protein lysates were loaded on 4–12% Bolt gels (Thermo Fisher Scientific) and separated for 30 min (200 volts, 3000 mA). After the separation, the gels were transferred to PVDF membranes for 60 min (20 volts, 3000 mA) and blocked with a blocking solution (skim milk, 5%) for 1.5 h. The membrane was washed for 1 h, incubated with primary antibodies for 18 h at 4 °C, and incubated again with secondary antibodies for 1 h at RT. To visualize protein bands, we used a bio‐imaging analyzer (iBright imaging system, Thermo Fisher Scientific). For quantification, we used multi‐gauge software (Fujifilm Corporation, Tokyo, Japan).

### Immunohistochemistry (IHC)

The IHC experiment was performed according to our previous reports with minor modifications [16, 17]. The cortical brain organoids were fixed for 24 h at RT in 4% PFA and incubated for 72 h at RT in 30% sucrose in PBS, pH 7.4 for dehydration. Subsequently, the samples were cryopreserved, sectioned, and permeabilized with 0.3% Triton X‐100 in PBS for 20 min at RT. The slices were then blocked for 1 h at RT in 5% (w/v) BSA. The organoids were incubated at 4 °C overnight in primary antibodies in 5% (w/v) BSA. On day 2, the organoids were washed and incubated with secondary antibodies for 1 h at RT. The organoids were stained with DAPI (Sigma‐Aldrich, D9542) for 10 min at RT, washed, and mounted for imaging analysis. For imaging analysis, Thunder Imager Live Cell and 3D Assay (Leica, Wetzlar, Germany) were used, and imaris software 9.91 (Oxford Instruments Bitplane, Oxfordshire, UK) was used to perform surface generation (smooth 0.5 for MAP2, 0.2 for others) and masking functions (surface: MAP2) for quantification of colocalized expressions between the two targets such as NR3A with PSD95 or mGluR8 with PSD95.

## Results and discussion

### Overall designs for this study

**Fig. 1:**
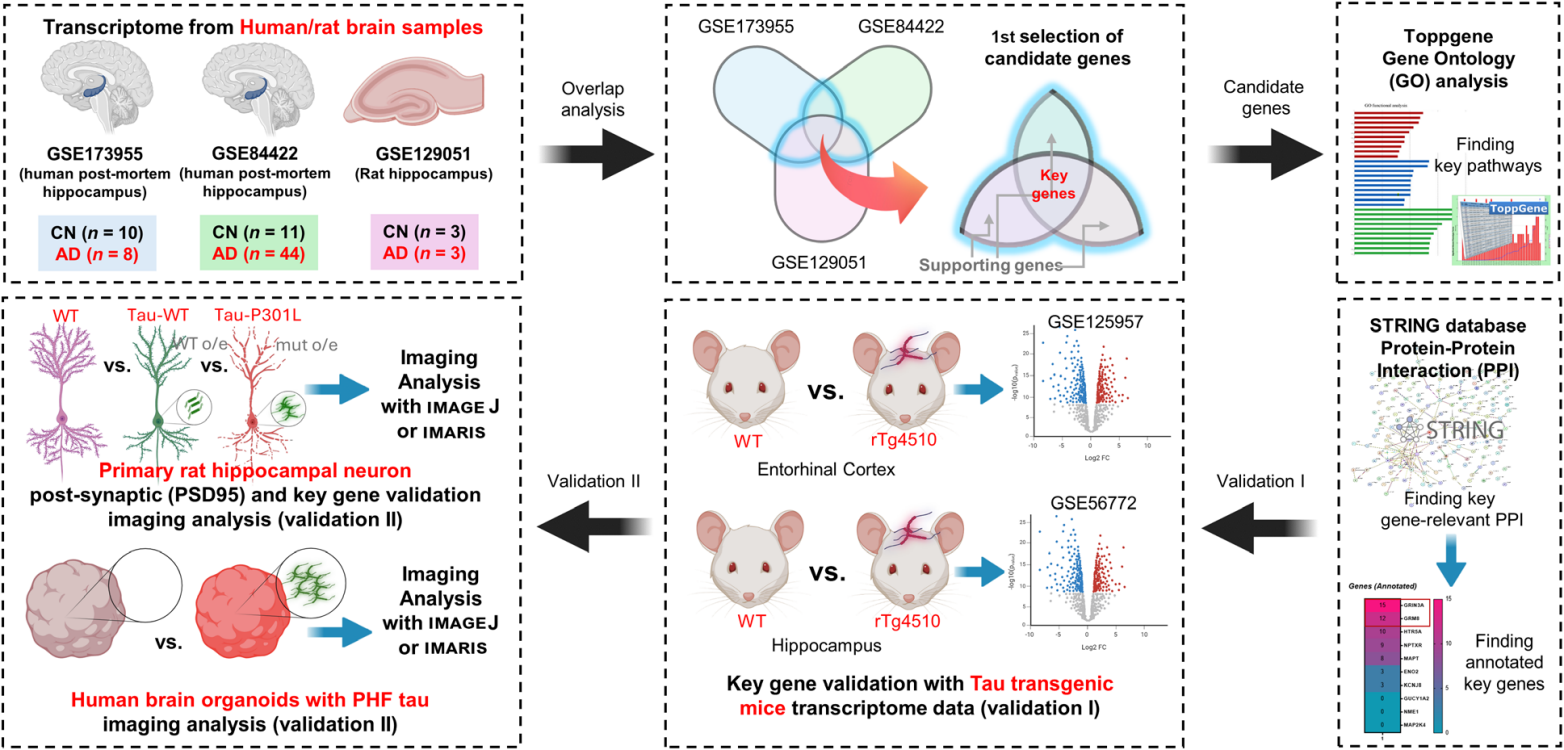
Experimental and analytical workflow of this study. For the selection of candidate genes, human postmortem hippocampus and rat hippocampus transcriptomic data from a publicly available database (GEO database) were used (GSE173955, GSE84422, and GSE129051). ToppGene and STRING databases were used for the selection of final genes. Final genes were validated by further transcriptome data with transgenic mice (validation I, GSE125957, GSE56772) and the experiments using primary rat hippocampal neurons and human brain organoids (validation II). AD, Alzheimer's disease; CN, cognitively normal control; PHF, paired helical filament.

We accessed NCBI GEO database to narrow down possible hippocampal biomarkers for AD. Numerous GEO datasets were examined, and three different datasets (GSE173955, GSE129051, and GSE84422) were collected. Transcriptome data were integrated to identify candidate genes by finding the intersection of these datasets. After narrowing down the candidate key and supporting genes (intersection between two datasets or all three datasets), the ToppGene database and STRING database were used for the selection of the final key and supporting genes. Final genes were validated in two steps; validation I compared transcriptome data (GSE125957, GSE56772) between wild‐type mice and tau transgenic (rTg4510) mice, and validation II was performed by using experimental models such as primary rat hippocampal neurons (WT vs. P301L tau mutant) and human brain organoids (Veh vs. PHF‐tau‐treated). The overall workflow of this study is graphically summarized in Fig. 1.

### Sorting out the candidate genes from multifarious human/rat postmortem transcriptome data

**Fig. 2:**
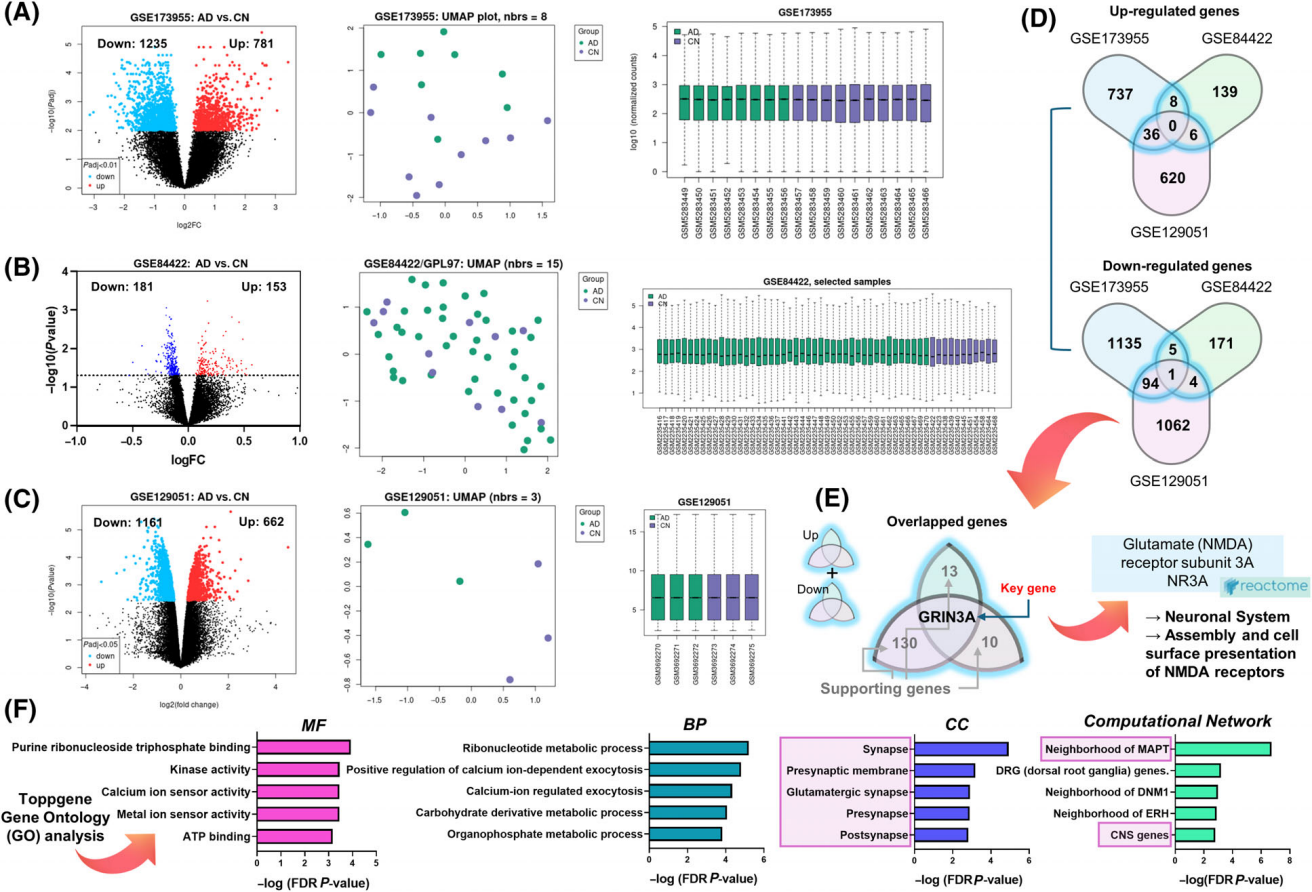
Characterization of RNA sequencing data and gene ontology (GO) analysis. (A–C) Characterization of transcriptome data (volcano plot, uniform manifold approximation and projection (UMAP), Boxplot) via GEO2R analysis. Volcano plots (left) show the number of upregulated and downregulated significant genes (*P*_adj._ < 0.05 for GSE173955 and GSE129051; *P*_raw_ < 0.05 for GSE84422). The UMAP graphs (middle) show the distribution of each sample. The boxplots (right) show the suitability for differential expression analysis. (D) Venn diagrams showing overlapped (intersection between two groups or all three groups) upregulated or downregulated genes. (E) GRIN3A was the only overlapped gene between all datasets (intersection between all three datasets). (F) ToppGene‐based GO analysis. The lavender‐colored boxes show the relationship between the overlapped genes and GO terms, especially synaptic regulation or MAPT‐related pathways. *P*‐values were false discovery rate (FDR)‐corrected. BP, biological process; CC, cellular component; MF, molecular function.

Since the hippocampus is a key brain region involved in learning and memory and its substantial association with AD, we accessed multifarious transcriptome datasets from the NCBI GEO database and tried to narrow down candidate hippocampal biomarkers for AD. We found three different hippocampal brain transcriptome datasets (GSE173955, human postmortem hippocampus, 10 cognitively normal (CN) participants and 8 patients with AD (AD); GSE84422, human postmortem hippocampus, 11 CN and 44 AD; GSE129051, rat hippocampus, 3 CN mice and 3 AD mice) and performed comparison analyses. We first identified upregulated and downregulated differentially expressed genes (DEGs) of each dataset (GSE173955, *n* = 1235 down and *n* = 781 up; GSE84422, *n* = 181 up and *n* = 153 down; GSE129051, *n* = 1161 up and *n* = 662 down) (Fig. 2A–C, left). For quality checks of datasets, UMAP graphs and boxplots were used. The UMAP graphs (Fig. 2A–C, middle) showed the distribution of each sample, and the boxplots (Fig. 2A–C, right) showed the suitability for differential expression analysis. Next, to narrow down candidate key genes and supporting genes, Venn diagrams were used. We identified 154 genes from the intersection between two datasets or all three datasets of both the up‐regulated Venn diagram and the downregulated Venn diagram (Fig. 2D). Interestingly, *GRIN3A*, glutamate ionotropic receptor NMDA‐type subunit 3A, was the only overlapped gene between all datasets (intersection between all three datasets). Since GRIN3A gene is highly associated with neuronal system and assembly and cell surface presentation of NMDA receptors according to the Pathway Browser of Reactome database (https://reactome.org), we speculated that downregulation of *GRIN3A* is linked with the synaptic dysfunction of patients with AD (Fig. 2E). We further performed ToppGene‐based GO analysis with ToppFun function with FDR‐corrected *P*‐values (*P* < 0.1). MF, BP, CC, and computational network terminologies were extracted, and significant terms were identified (Fig. 2F). We confirmed that the genes were highly associated with synaptic functions such as pre‐ or postsynapse, glutamatergic synapse, and synaptic membrane. Moreover, the genes were linked with the network of MAPT (microtubule‐associated protein tau) and central nerve system (CNS) indicating that *GRIN3A* and candidate supporting genes are related to hippocampal tau‐mediated dysfunction of AD.

### STRING PPI mapping revealed the key and supporting genes

**Fig. 3:**
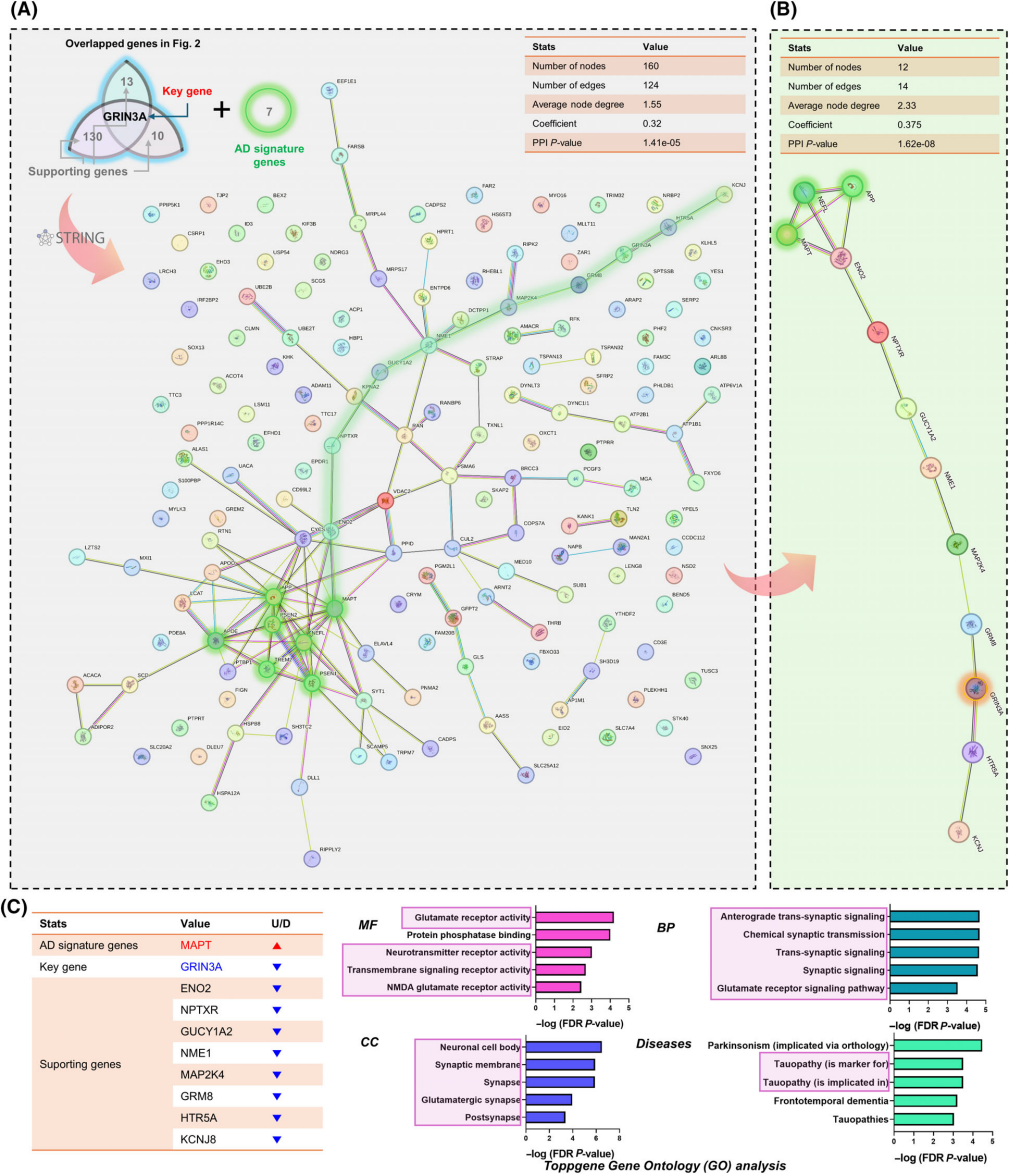
Protein–protein interaction (PPI) analysis (STRING database) and selection of final key genes with gene ontology (GO) analysis. (A, B) The PPI network mapping result with GRIN3A, supporting genes, and Alzheimer's disease (AD) signature genes. Seven AD signature genes and GRIN3A‐related interactions were green‐highlighted. (C) A selected key gene (GRINS3A) and supporting genes (8 genes) with MAPT AD signature gene were used for ToppGene‐based GO analysis and annotated genes and their counts for GO terminologies. *GRIN3A* and *GRM8* were the top two genes for GO terminologies. *P*‐values were false discovery rate (FDR)‐corrected. BP, biological process; CC, cellular component; MF, molecular function.

**Fig. 4:**
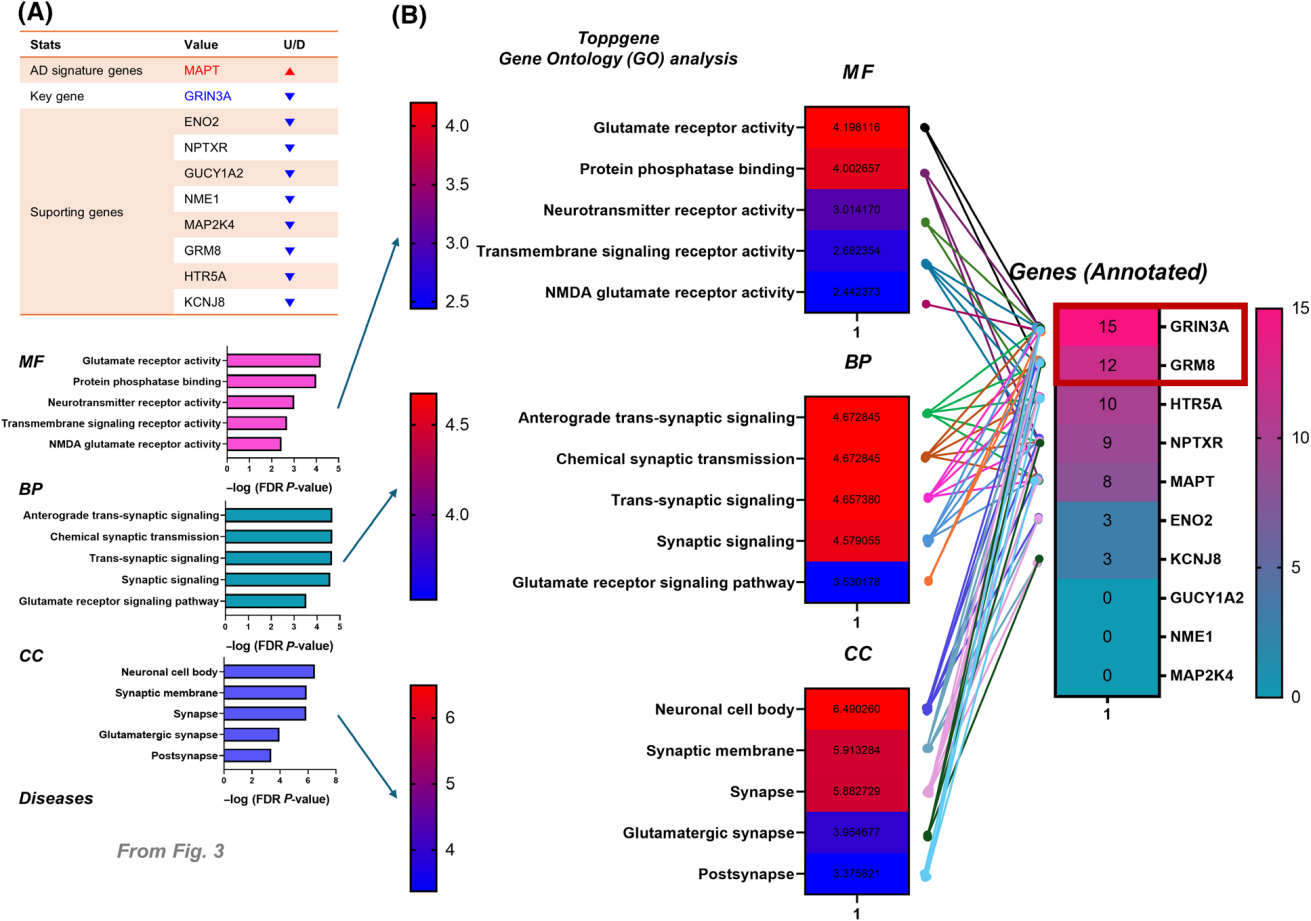
Detailed gene annotation information. (A) The same data in Fig. 2. A selected key gene (*GRIN3A*) and supporting genes (8 genes) with MAPT AD signature gene were used for ToppGene‐based gene ontology (GO) analysis. *P*‐values were false discovery rate (FDR)‐corrected. BP, biological process; CC, cellular component; MF, molecular function. (B) Detailed information for annotated genes and their counts for GO terminologies. *GRIN3A* (15 terminologies) and GRM8 (12 terminologies) were the top two genes.

To identify *GRIN3A*‐related pathways and their PPI regarding the progression of AD, STRING PPI analysis was performed. With GRIN3A and supporting 153 genes, 7 AD signature genes (*APP*, *PSEN1*, *PSEN2*, *MAPT*, *APOE*, *TREM2*, and *NEFL*) were used as inputs together (Fig. 3A). Interestingly, we found a PPI cluster (around *GRIN3A*) which is linked with 3 AD signature genes (*MAPT*, *APP*, and *NEFL*) (Fig. 3B). Since ToppGene GO already showed the possibility of a relationship with the MAPT network (Fig. 2F, right), we selected MAPT as a target for validation among 3 AD signature genes, with the nine key and supporting genes (Fig. 3C, left table). We performed ToppGene GO analysis with these 10 genes and confirmed numerous significant terms that are related to *GRIN3A*‐mediated pathways, such as glutamate receptor activity, neurotransmitter receptor activity, transmembrane signaling receptor activity, NMDA glutamate receptor activity, anterograde trans‐synaptic signaling, postsynapse, and synaptic membrane (Fig. 3C, right graphs). To determine final targets for validation with the experimental model, checking gene annotations for each GO term was required (Fig. 4A). We found that *GRIN3A* and *GRM8* were the top two annotated genes for each GO term and determined to perform further validations of two genes (Fig. 4B).

### Tau transgenic mice (rTg4510) showed GRIN3A deficiency

Before performing experimental validations, we further checked other possible publicly available datasets that are associated with tau mutation in AD. We selected two datasets (GSE125957 and GSE56772) which used tau transgenic mice, rTg4510 (P301L mutant) (Fig. S1A). For GSE125957, we compared the expression levels of *GRIN3A*, *GRIN3B*, *GRIN1*, and *GRM8* (Fig. S1B). We confirmed that *GRIN3A* and *GRIN1*, which forms a di‐heteromeric NMDA receptor with *GRIN3A* [19], showed downregulated tendency in rTg4510 (TG) across all age groups (2, 4, 6, 8 months) (Fig. S1B, upper left and right). However, *GRIN3B* was not significantly different between WT (wild type) vs. TG (rTg4510) indicating that tau‐induced deficiency of the *GRIN3* family is a *GRIN3A*‐specific phenomenon (Fig. S1B, lower left). We also confirmed that the expression levels of *GRM8* were significantly reduced in 4 and 6 months of TG (Fig. S1B, lower right). For further validation through the GEO dataset, GEO2R analysis was performed with the GSE56772 dataset. Although the comparison of *GRIN3A* was not available since *GRIN3A* was not annotated (not listed) for the GSE56772 dataset, *GRIN1* and *GRM8* were significantly decreased in TG, but *GRIN3B* was not decreased in TG, like the results from GSE125957 dataset. Thus, we identified that tau transgenic mice showed *GRIN3A* and *GRM8* deficiency, and further experiments by using P301L mutant for validation were needed.

### Hippocampal NR3A deficiency in tau‐induced primary rat hippocampal neurons

**Fig. 5:**
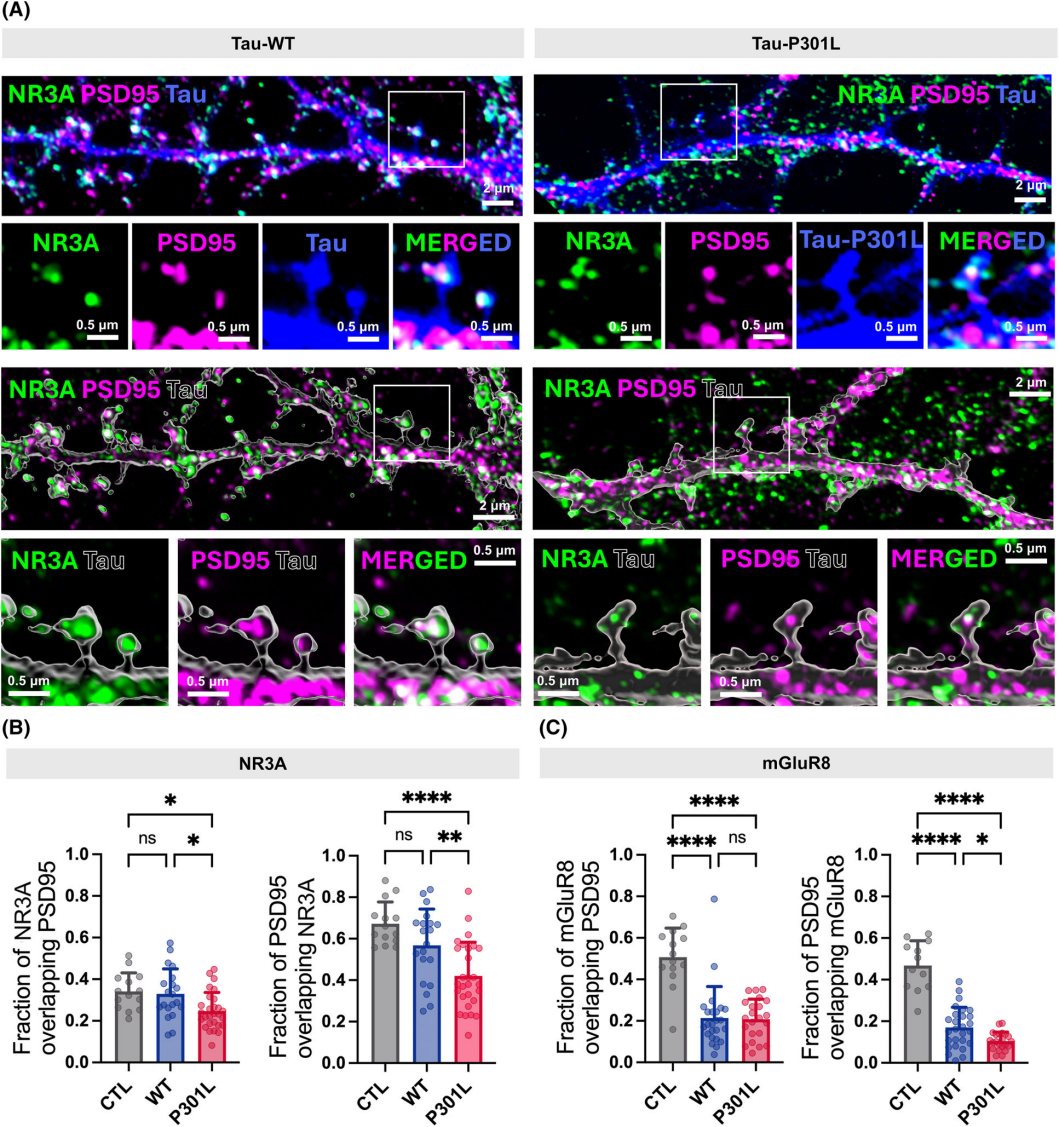
Experimental validation of hippocampal tau‐induced NR3A deficiency by using primary rat hippocampal neurons (A) Representative images of primary hippocampal neurons labeled for NR3A (Green, protein translated from GRIN3A) and PSD95 (Magenta). Cultured hippocampal neurons were transfected with WT Tau‐GFP or P301L Tau‐GFP, followed by immunocytochemistry using specific antibodies against NR3A and PSD95. The lower panels show enlarged single spines. 3D volume‐rendered images using imaris. Scale bar: 2 and 0.5 μm. (B, C) Quantification of Manders' coefficients of NR3A and PSD95. For quantification, regions of interest (ROIs) were selected on dendrites containing at least 8 spines from 3 to 5 individual neurons. Each group included 12–28 ROIs obtained from 3 independent cultures. Data shown as mean + SD. One‐way ANOVA followed by Tukey's multiple comparisons test, *P < 0.05, **P < 0.01 and ****P < 0.0001.

To investigate whether NR3A (protein name encoded by *GRIN3A*; NMDA‐type glutamate receptor subunit 3A) shows lower expression levels within dendritic spines, we performed immunocytochemistry on primary cultured hippocampal neurons using specific antibodies against NR3A, mGluR8 (protein name encoded by GRM8; metabotropic glutamate receptor 8), and PSD95. Subsequently, we captured images using Airyscan‐based super‐resolution microscopy (Fig. 5). As expected, NR3A was highly enriched in dendritic spines in control and Tau‐WT‐expressed neurons (Fig. 5A,B). Interestingly, the proportion of NR3A overlapping PSD95 and *vice versa* was significantly reduced only in neurons expressing the pathogenic Tau‐P301L variant. These findings suggest that the decreased PSD enrichment of NR3A may be a consequence of Tau‐P301L pathology. On the other hand, the colocalization of mGluR8 with PSD95 (Fig. 5C) shows strikingly reduced in both neurons expressing Tau‐WT and Tau‐P301L which implies that mGluR8 dysfunction might extend beyond Tau‐P301L specific pathology and potentially reflects a more general effect of Tau overexpression on postsynaptic function.

### NR3A deficiency in the PHF‐tau‐treated iPSC‐derived human brain organoids

**Fig. 6:**
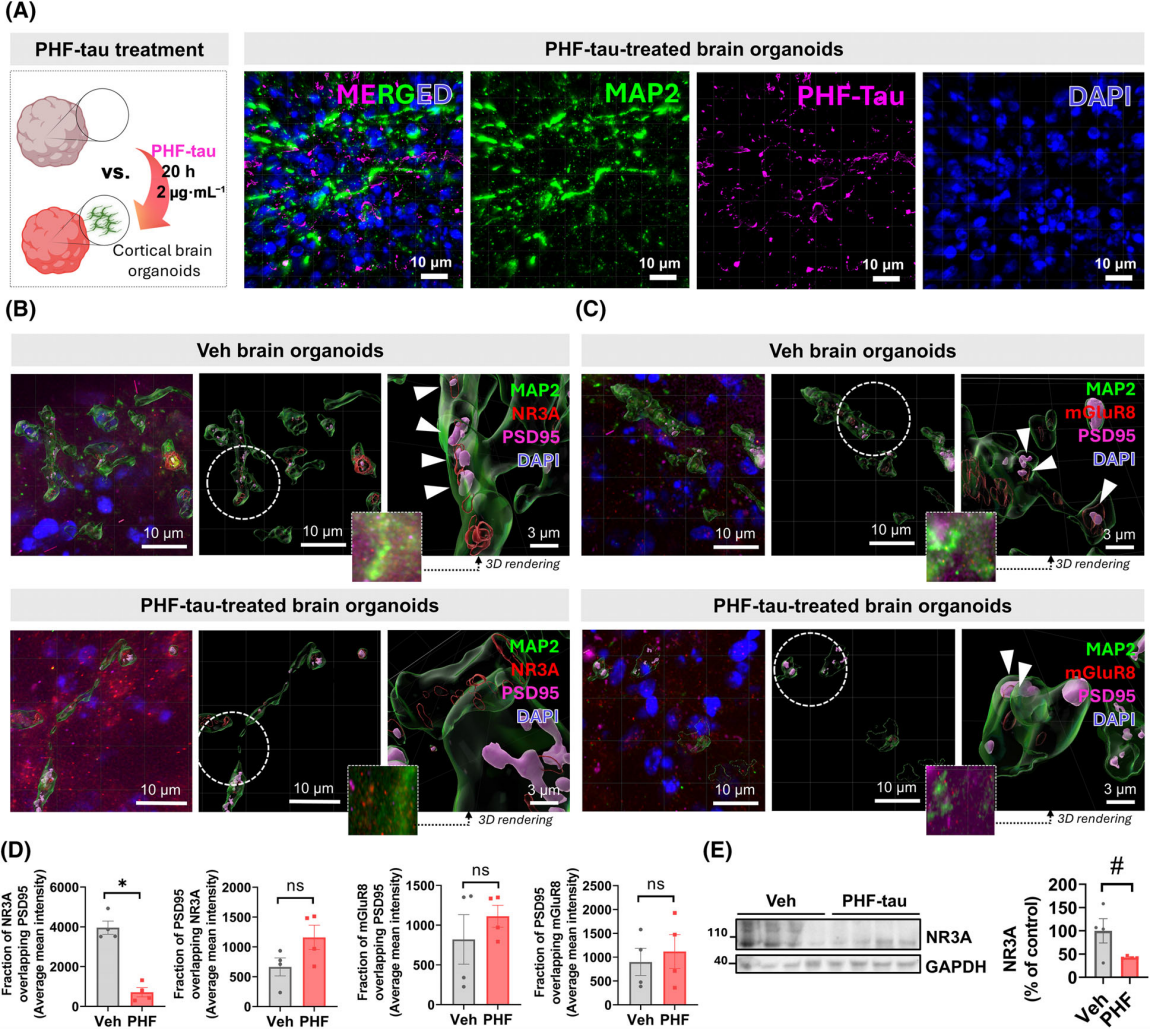
Experimental validation of tau‐induced NR3A deficiency by using human iPSC‐derived brain organoids. (A) Representative images of paired helical filament (PHF)‐tau‐treated brain organoids. The organoids were labeled for MAP2 (green), PHF‐tau (magenta), and DAPI (blue). Scale bar = 10 μm. (B) Representative 3D view images of IMARIS 3D rendering to compare fractions of NR3A or PSD95 overlapped with each other. The organoids were labeled for MAP2 (green), NR3A (red), PSD95 (magenta), and DAPI (blue). White arrows show the positions colocalized between PSD95 and NR3A. The dotted circles show the magnified positions. *n* = 4 slides for each group. Scale bar = 10 or 3 μm. (C) Representative 3D view images of imaris 3D rendering to compare fractions of mGluR8 or PSD95 that overlapped with each other. The organoids were labeled for MAP2 (green), mGluR8 (red), PSD95 (magenta), and DAPI (blue). White arrows show the positions colocalized between PSD95 and mGluR8. The dotted circles show the magnified positions. *n* = 4 slides for each group. Scale bar = 10 or 3 μm. (D) Quantification data of (B) and (C). *P*‐values by Mann–Whitney test (*P < 0.05). *n* = 4 slides for each group. (E) Western blot analysis to compare the total levels of NR3A between PHF‐tau‐treated organoids and vehicles. *P*‐values by Welch's *t*‐test (^#^ *P* = 0.1).

Similar to Fig. 5, we also performed IHC to investigate whether NR3A and mGluR8 show lower expression levels within dendritic spines (Fig. 6). We treated PHF‐tau (2 μg·mL^−1^, 20 h) to induce tauopathy in brain organoids and confirmed that extracellular PHF‐tau aggregates existed in brain organoids (Fig. 6A). Subsequently, we captured images using Thunder microscopy and confirmed that the proportion of NR3A overlapping PSD95 was significantly reduced only in neurons expressing the pathogenic PHF‐tau‐treated organoids (Fig. 6B,D). However, unfortunately, we could not find any significant differences in the levels of mGluR8 between PHF‐tau‐treated organoids and vehicles (Fig. 6C,D). Moreover, we performed WB analysis and confirmed that there is a decrease in the levels of total NR3A proteins in PHF‐tau‐treated organoids compared to vehicles (Fig. 6E). These results indicate that there were slight variations between species (rat vs. human), demonstrating that GRIN3A is a more significant biomarker that is not affected by species‐specific influences.

## Conclusions

The GRIN3A gene encodes the NR3A subunit of NMDA receptors, a key component of glutamatergic neurotransmission in the CNS. NR3A modulates NMDA receptor activity, thereby influencing synaptic plasticity, neuronal connectivity, and ultimately, cognitive function. Disruptions in *GRIN3A* expression or function may lead to aberrant glutamate signaling, potentially contributing to the pathophysiology of various neurological disorders, including schizophrenia, autism spectrum disorder, and intellectual disability [20, 21, 22, 23]. Studies in mouse models further suggest a direct association between phosphorylated tau and altered NMDA receptor function, even in the absence of amyloid beta (Aβ) pathology [24]. Building on this, our study also implicates tau in the mechanisms underlying abnormal network activity within the hippocampal circuit.

The *GRM8* gene encodes the metabotropic glutamate receptor 8 (mGluR8), a G protein‐coupled receptor belonging to the mGluR family. mGluR8 expression is prominent throughout the CNS, with particularly high levels observed in the hippocampus, cortex, and cerebellum [25]. This receptor plays a diverse role in CNS function, including regulating synaptic plasticity, cognitive function, and mood. Due to its involvement in these critical processes, mGluR8 represents a potential therapeutic target for various neurological disorders, including AD [26, 27, 28]. Although mGluR8 and NR3A are components of different receptor systems, we believe they can interact within the broader context of glutamatergic synaptic functions. This is supported by their colocalization with PSD95 and their association with decreased postsynaptic function when neurons are exposed to PHF‐tau (Fig. 5).

Such a multidimensional analysis is indispensable for elucidating the intricate molecular mechanisms of AD [29]. The heterogeneity of AD, driven by a myriad of genetic, environmental, and epigenetic factors, necessitates a comprehensive strategy to distill common molecular signatures and disrupt biological processes [30]. Therefore, by leveraging diverse transcriptome datasets, from sources such as GSE173955, GSE129051, and GSE84422, our approach ensured a more comprehensive and robust identification of critical genes and pathways implicated in AD. We thought that integrating gene expression profiles from multiple sources mitigates interindividual and technical variability inherent in individual studies, enhancing the reliability and validity of our findings. Also, we thought that this rigorous approach facilitates the identification of genes and pathways consistently altered in AD, thereby providing a more precise and holistic understanding of the disease's molecular underpinnings. Furthermore, accurately pinpointing specific molecular targets through this integrative approach is paramount for developing efficacious therapeutic interventions. By elucidating the genes and pathways most pivotal to AD pathology, our research highlights these targets for innovative drug development and therapeutic strategies. For instance, identifying key genes such as GRIN3A and their associated pathways, including NMDA receptor signaling, unveils new avenues for targeted therapies aimed at modulating these pathways to mitigate or arrest AD progression.

In sum, by utilizing various databases (five transcriptome GEO datasets, STRING database, ToppGene database) and experimental validation model such as primary rat hippocampal neuron and iPSC‐derived human brain organoids, we identified a novel tauopathy biomarker *GRIN3A* in AD. Moreover, we believe that our integrated approach, which combines analyses using publicly available multiple databases with diverse experimental validations, has the potential to advance precision medicine therapies in the era of bioinformatics.

## Conflict of interest

The authors declare no conflict of interest.

### Peer review

The peer review history for this article is available at https://www.webofscience.com/api/gateway/wos/peer‐review/10.1002/2211‐5463.13904.

## Author contributions

J‐CP: Conceptualization. S‐EL, SP, RK, TL, WJY, and J‐CP: Investigation. S‐EL, SP, RK, and J‐CP: Methodology and Visualization. S‐EL, SP, SC, and J‐CP: Resources. S‐EL, SP, and J‐CP: Writing‐original draft. S‐EL, SC, and J‐CP: Writing‐review and editing.

## Supporting information


**Fig. S1.** Transcriptome databases from Tau transgenic mice (rTg4510) reveal *GRIN3A* deficiency in Alzheimer's disease.

## Data Availability

The datasets used and/or analyzed during the current study are available from the corresponding author upon reasonable request.
