# Supplementary material for: Hippocampal tau‐induced GRIN3A deficiency in Alzheimer's disease
Source: FEBS Open Bio. 2024 Oct 13;14(12):2059–71. doi: 10.1002/2211-5463.13904 (PMC11609574; doi:10.1002/2211-5463.13904)
Supplement: Supplementary file 1 — Fig. S1. Transcriptome databases from Tau transgenic mice (rTg4510) reveal GRIN3A deficiency in Alzheimer's disease. [file FEB4-14-2059-s001.pdf]

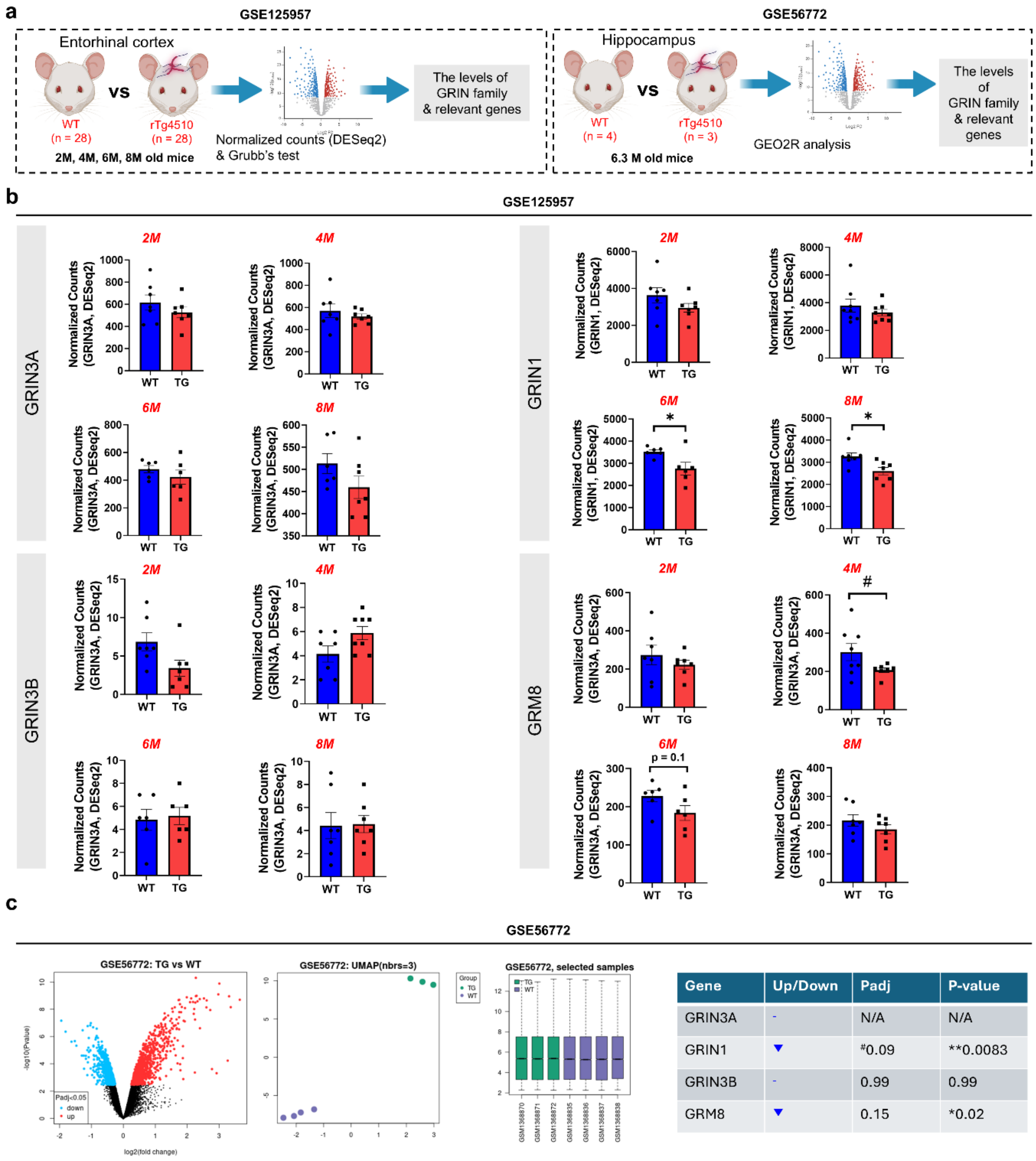

Gene	Up/Down	Padj	P-value
GRIN3A	-	N/A	N/A
GRIN1	▼	#0.09	**0.0083
GRIN3B	-	0.99	0.99
GRM8	▼	0.15	*0.02

Supplementary Figure 1. Transcriptome databases from Tau transgenic mice (rTg4510) reveal *GRIN3A* deficiency in Alzheimer's disease (a) Two GEO databases (GSE125957 and GSE56772) using rTg4510 (TG) and wild-type (WT) mice. **(b)** Comparison of the levels of GRIN3A-related genes (*GRIN3A, GRIN3B, GRIN1, GRM8*) between TG and WT mice (GSE125957). $^{\#}p < 0.1$, $^{*}p < 0.05$, $^{**}p < 0.01$. p-values by independent t-test. **(c)** The levels of *GRIN1* and *GRM8* were significantly decreased in TG compared to WT mice (GSE56772). The comparison of *GRIN3A* was not available since *GRIN3A* was not annotated (not listed) for the GSE56772 dataset. $^{\#}p < 0.1$, $^{*}p < 0.05$, $^{**}p < 0.01$. p-values by GEO2R analyzer (P-values after adjustment for multiple testing or raw p-values).
